# In silico-prediction of protein–protein interactions network about MAPKs and PP2Cs reveals a novel docking site variants in *Brachypodium distachyon*

**DOI:** 10.1038/s41598-018-33428-5

**Published:** 2018-10-10

**Authors:** Min Jiang, Chao Niu, Jianmei Cao, Di-an Ni, Zhaoqing Chu

**Affiliations:** 1 Shanghai Key Laboratory of Plant Functional Genomics and Resources, Shanghai Chenshan Botanical Garden, Shanghai, China; 2 Shanghai Chenshan Plant Science Research Center, Chinese Academy of Sciences, Shanghai, China; 3 School of Ecological Technology and Engineering, Shanghai Institute of Technology, Shanghai, China

## Abstract

Protein-protein interactions (PPIs) underlie the molecular mechanisms of most biological processes. Mitogen-activated protein kinases (MAPKs) can be dephosphorylated by MAPK-specific phosphatases such as PP2C, which are critical to transduce extracellular signals into adaptive and programmed responses. However, the experimental approaches for identifying PPIs are expensive, time-consuming, laborious and challenging. In response, many computational methods have been developed to predict PPIs. Yet, these methods have inherent disadvantages such as high false positive and negative results. Thus, it is crucial to develop in silico approaches for predicting PPIs efficiently and accurately. In this study, we identified PPIs among 16 BdMAPKs and 86 BdPP2Cs in *B. distachyon* using a novel docking approach. Further, we systematically investigated the docking site (D-site) of BdPP2C which plays a vital role for recognition and docking of BdMAPKs. D-site analysis revealed that there were 96 pairs of PPIs including all BdMAPKs and most BdPP2Cs, which indicated that BdPP2C may play roles in other signaling networks. Moreover, most BdPP2Cs have a D-site for BdMAPKs in our prediction results, which suggested that our method can effectively predict PPIs, as confirmed by their 3D structure. In addition, we validated this methodology with known Arabidopsis and yeast phosphatase-MAPK interactions from the STRING database. The results obtained provide a vital research resource for exploring an accurate network of PPIs between BdMAPKs and BdPP2Cs.

## Introduction

Protein kinases or phosphatases in eukaryotic cells are main basics of signal transduction mechanisms by catalyzing covalent addition or subtraction of phosphate groups to serine and threonine/tyrosine residues in their substrates, which causes the quick and reversible modification of proteins and thus regulates plant living situations to adapt to their environment rapidly and precisely^1^. In particular, mitogen-activated protein kinase (MAPK, also called MPK) is a crucial protein kinase plays vital role in converting environmental and developmental signals into distinct nuclear responses^2,3^. MAPKs are activated by their specific activator MAPK kinases (MAPKKs, MKKs) through double phosphorylation on both conserved threonine (T) and tyrosine(Y) residues located in the kinase activation loop^4,5^. In contrast, inactivation of MAPKs is induced by dephosphorylation of cognate residues by various tyrosine phosphatases^6^, serine/threonine phosphatases(e.g.PP2C)^7^ and/or dual-specificity phosphatases (DUSPs)^8^.

The formation of complex between MAPK and its connate activator, substrate, scaffold or inactivator is normally achieved through specific docking interactions^9^. The docking interactions increase the efficiency of all the enzymatic reactions and may help to regulate the specificity of molecular recognition^10^. It was discovered that MAPKs contain a common docking domain (CD domain) that is featured by a cluster of negatively charged amino acids in the C-terminal region outside the catalytic domain that binds the basic residues at the N terminus of the docking site (D-site) in MAPK-interaction proteins^11,12^. Such D-sites has more consecutive positively charged amino acids and promote binding specificity and high affinity interactions with cognate MAPKs. D-sites are also found in MAPK regulating proteins such as MKKs^13,14^, scaffold proteins and MAPK phosphatases and substrates^10^. The D-sites in these proteins consist of a cluster of basic residues followed by a hydrophobic sub-motif containing Leu, Ile or Val separated by one residue (R/K_1–3_-X_1–6_- *φ*-X-*φ*) (*φ* is any hydrophobic residue)^15,16^. The specificity of MAPK docking interactions may be determined by the two hydrophobic residues at the distal end of the D-site in MAPKKs^17^. However, by comparing the current and previous structures, we found that MAPK have actually three (not two) hydrophobic pockets on this surface, which collectively form a “docking groove”, that bind the motif LxLxL/I^17^. Of course, it is least critical for MAPK binding about the most C-terminal hydrophobic residue in the LxLxL/I motif^18,19^. Furthermore, MAPK phosphatase MKP-1 harbor another type of MAPK-docking site named the DEF motif ^20^. Notably, D-sites bind to acidic residues in the CD domain of MAPKs, while DEF motifs interact with a hydrophobic pocket that is only exposed upon MAPK activation^21^. Moreover, the other one atypical MAPK-docking site within Msg5 named the IYT motif mediates the interaction of Slt2 and Mlp1^22,23^.

Naturally, the precondition of kinase-substrate reaction is the interaction between MAPK and cognate target proteins. However, it is difficult to identify the PPIs about these proteins, primarily because MAPK-substrate interactions are much transient and unstable. The identification of PPIs about their critical roles is a great challenge of unravel many interactomes for deciphering the molecular mechanisms and further providing insight into numerous physiological and pathological processes^24^. Consequently, the construction of dynamic PPI networks is a critical step towards better understanding biological function of relevant proteins. Recently, many experimental technologies have been proposed for the large-scale PPIs detection, for example, two-hybrid-based screens^25^, protein chips^26^, mass spectrometry^27^, bimolecular fluorescent complementation (BiFC)^28^ and phage display. However, these methods have itself inherent limitations such as high false positive rate and very low coverage^29,30^. Hence, more and more computational methods, such as evolutionary information and physicochemical characteristics method^31^, alignments of multiple sequences^32^ or weighted sparse representation model^33^, and so on, have been proposed to predict PPIs efficiently and accurately. While in silico-prediction of PPIs using “Docking” strategy about MAPK has been developed in this study, which can avoid these disadvantages mentioned above.

The above observations clearly demonstrate that MAPK-PP2C docking is crucial for efficient signal transmission. However, less is known about the role of MAPK-PP2C docking in specificity and the identification of D-site in PP2C that is only found in subgroup B PP2C^34–36^. In order to better understand the PPIs about MAPKs and PP2Cs, we have generated a PPI network based on docking approach to predict the interactions about MAPKs and PP2Cs in *Brachypodium distachyon*. And further analysis also has performed to study the key basis about MAPK-docking on PP2C proteins. In general, the network of PPIs can provide novel insights into the molecular mechanism and the clues of function about MAPKs and PP2Cs in *B. distachyon*.

## Results

### Prediction of interaction pairs

Networks of PPIs supply a framework for understanding the biological processes and can give insights into the molecular mechanism inside the cell. Thus the scholarship of PPIs promotes the understanding of biological mechanisms. MAPK is regulated through gene expression between cell receptor and cell response by transcription factions. To better elucidate the molecular mechanism, our knowledge no work in the literature has been executed regarding the interaction prediction of PP2Cs with MAPKs in *B. distachyon*. MAPKs are prominent components of protein phosphorylation cascades transduce extracellular signals to plant defense responses. Thus this paper identifying the interacting MAPKs with PP2Cs of *B. distachyon* which is relate to disease signaling process resorting to docking approach. Docking studies is a method which predicts two proteins (PP2C and MAPK) when bound to each other to a complex, the more stable structure (less global energy) higher the probability of their interaction.

**Figure 1:**
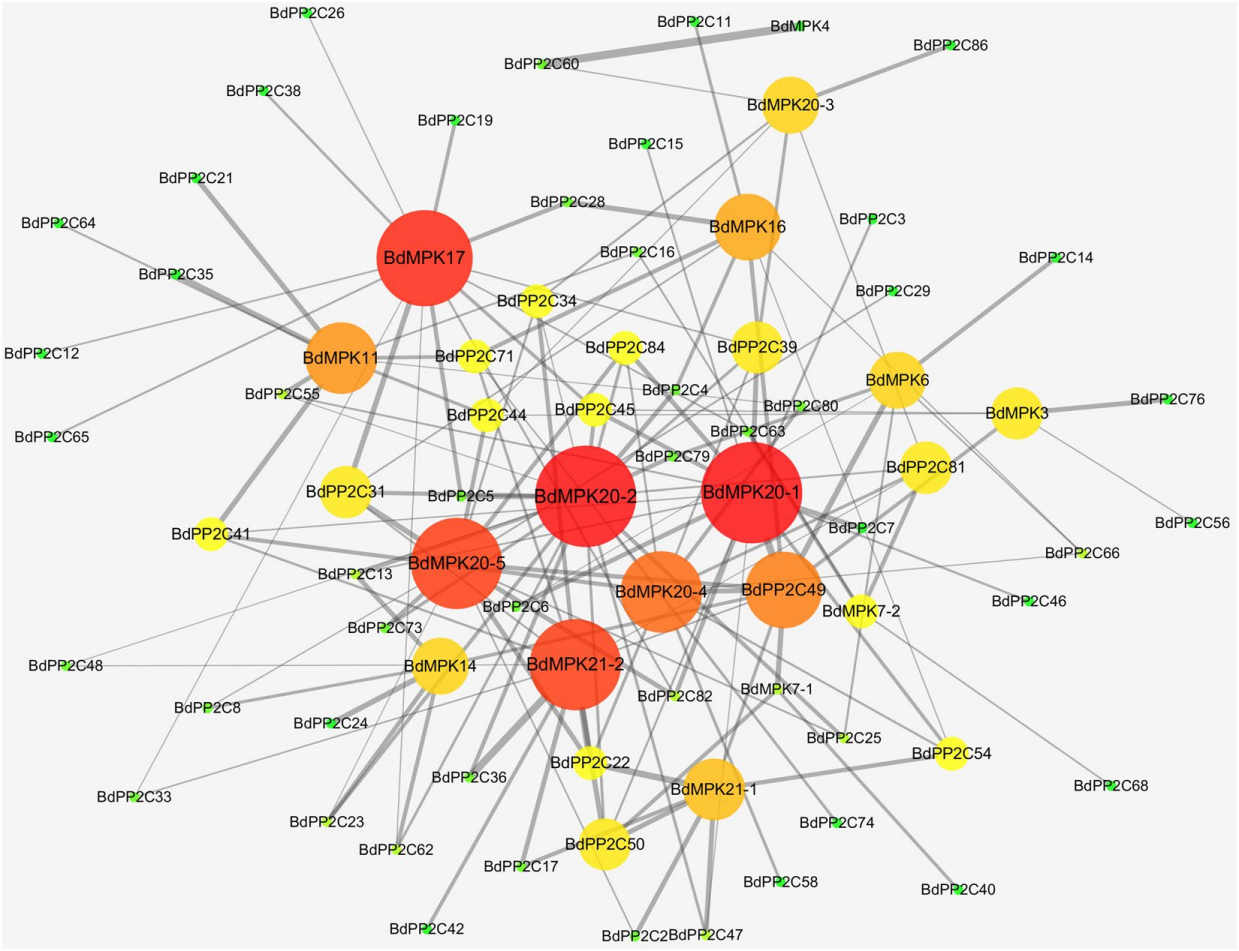
Visualization for the predicted results of PPIs between BdPP2Cs and BdMAPKs was used by the Cytoscape tool. Each node represents a protein and each line refers an interaction. Line thickness reflects the strength of PPIs. Font size and circle color indicated the number of PPIs. Our results showed that there are 143 pairs of potential PPIs. The network is generated using the Cytoscape tool.

We defined that the absolute value of the lowest energy (AVLE) of two proteins is higher than 65.74 that is the maximum value of the interaction between all subgroup B PP2Cs and MPKs, which means them interacting with each other. Docking studies revealed that 96 from 1376 pairs of PPIs, that is 6.98℅, exhibit higher possibility of interaction including all MAPKs and 49 PP2Cs (Fig. 1, Table S1). The results suggested that PP2C may be connected with other signaling pathway in which PP2C also plays vital roles about negative regulator^36,37^. Moreover, not all of BdMAPKs and BdPP2Cs are only connected into a single network component (Fig. 1). Some proteins show highly correlated while others do not. For example, BdMAPK20-1, BdMAPK20-2, BdMAPK20-4 and BdMAPK20-5 interact with more BdPP2Cs than others. Of course, there were also some BdPP2Cs showed stronger interaction with BdMAPKs in the chart, including BdPP2C22, BdPP2C31, BdPP2C34, BdPP2C39, BdPP2C41, BdPP2C45, BdPP2C49, BdPP2C50, BdPP2C71 and BdPP2C84(Fig. 1, Table S1). These results indicated that our method could be used to predict the PPIs between BdPP2Cs and BdMAPKs, which were effortless and timesaving in silico “Docking” strategy.

### Identification of putative D-site in BdPP2Cs

Docking interaction is the precondition of protein molecular recognition and enzymatic reaction. Except subgroup B PP2C, no MAPK-docking sites have been reported. To investigate the correlation of docking site for MAPK with the prediction results, we further analyzed the sequence features of PP2C such as the existence or position of putative docking site. In order to search for the sequence that conform the rules of D-site in PP2C using hidden Markov model, we have derived a list of potential novel D-site (PP2C) from *B. distachyon* which also showed interaction with BdMAPK through docking studies (Table 1). There are 34 BdPP2C proteins which also show 73.96% PPIs harboring D-site in identified interaction with BdMAPK (Table 1). D-finder assigned a Viterbi probability score of 2.85E-22 to a putative D-site (core sequence: **RR**G**RRR**PDILTI) that it found in residues 29–40 of BdPP2C55 (Table 1). It could be hypothesized that the amino acids surrounding the minimal D-site motif contribute to MAPK function. The results indicated that the prediction PPIs have the structural basis of recognition each other and also clearly revealed the complexity of BdMAPK interaction with several PP2C triggered in response to diverse upstream stimuli. Of course, number of novel candidate BdMAPK negative regulators was predicted and need to be confirmed by *in vitro* assay.

**Table 1:** Docking sites of potential interacting BdPP2Cs identified for MAP kinases *in B. distachyon*.

Docking protein	Docking site	MPK	Score
BdPP2C2	KGPRRRHVAPAALP	MPK21-1	1.84E-27
BdPP2C3	RRAPAAAFVAAI	MPK20-1	6.79E-28
BdPP2C6	RRGKGRNRLEM	MPK20-1	1.80E-25
BdPP2C7	REKARLPPALPL	MPK20-1	1.01E-27
BdPP2C21	RKIFIKLDF	MPK11	9.56E-28
BdPP2C22	RRSKTFLVL	MPK20-4,MPK20-5,MPK21-1,MPK21-2	3.16E-28
BdPP2C23	KSKKGEDFTLLV	MPK14,MPK20-2	8.41E-26
BdPP2C24	KRGEDYFLVKP	MPK3,MPK14	4.63E-29
BdPP2C25	RRSLSCKA	MPK6,MPK20-2	8.27E-30
BdPP2C28	KNHKKKRVVA	MPK16,MPK17	1.11E-25
BdPP2C31	RRSRFSPLRA	MPK17,MPK20-2,MPK20-5	4.43E-28
BdPP2C34	KRLGVRHPLKY	MPK20-3,MPK20-5,MPK21-2	2.15E-27
BdPP2C35	RRPEMEDAAAVL	MPK11	1.44E-28
BdPP2C36	RRRLRADAGA	MPK20-2,MPK21-2	9.59E-29
BdPP2C38	RRWQEAVAL	MPK17	5.58E-26
BdPP2C39	RRNRRLDAV	MPK20-1,MPK20-2,MPK20-3,MPK20-4	2.48E-30
BdPP2C41	RLRRALASLPL	MPK11,MPK20-4,MPK21-2	1.37E-28
BdPP2C42	KKNMVGTLIYMA	MPK21-2	1.99E-27
BdPP2C45	KKTDLDLLDA	MPK17,MPK20-1,MPK20-2	6.21E-28
BdPP2C46	RRLGRTASAAA	MPK20-1	7.43E-25
BdPP2C47	RRRRRLEMRRFRL	MPK20-2,MPK21-1	1.20E-28
BdPP2C49	RRAAAWLL	MPK3,MPK6,MPK7-1,MPK14,MPK16,MPK20-1,MPK20-4,MPK20-5,MPK21-1	6.20E-28
BdPP2C54	SRKVRVPL	MPK20-1,MPK20-4,MPK21-1	7.82E-28
BdPP2C55	RRRPDILTI	MPK11	2.85E-22
BdPP2C58	RSRKGADAA	MPK20-4	6.87E-28
BdPP2C60	KKGVNQDAMVVW	MPK4	3.34E-25
BdPP2C71	RRRSQEDRAVCAL	MPK11,MPK16,MPK21-2	6.11E-28
BdPP2C73	KRRPSMLVIPV	MPK20-2,MPK20-5	4.70E-27
BdPP2C74	KRLKGAATSI	MPK20-2	1.55E-30
BdPP2C76	KKNLLDNVL	MPK3	1.05E-28
BdPP2C79	RKGQIFCGV	MPK6,MPK20-2	3.89E-28
BdPP2C81	RARKGEDYALLKL	MPK7-2,MPK20-4	2.47E-25
BdPP2C84	RKTLYL	MPK20-1,MPK20-2,MPK20-5	4.25E-28
BdPP2C86	KRASMEDFY	MPK20-3	1.00E-40

### Model Validation

**Figure 2:**
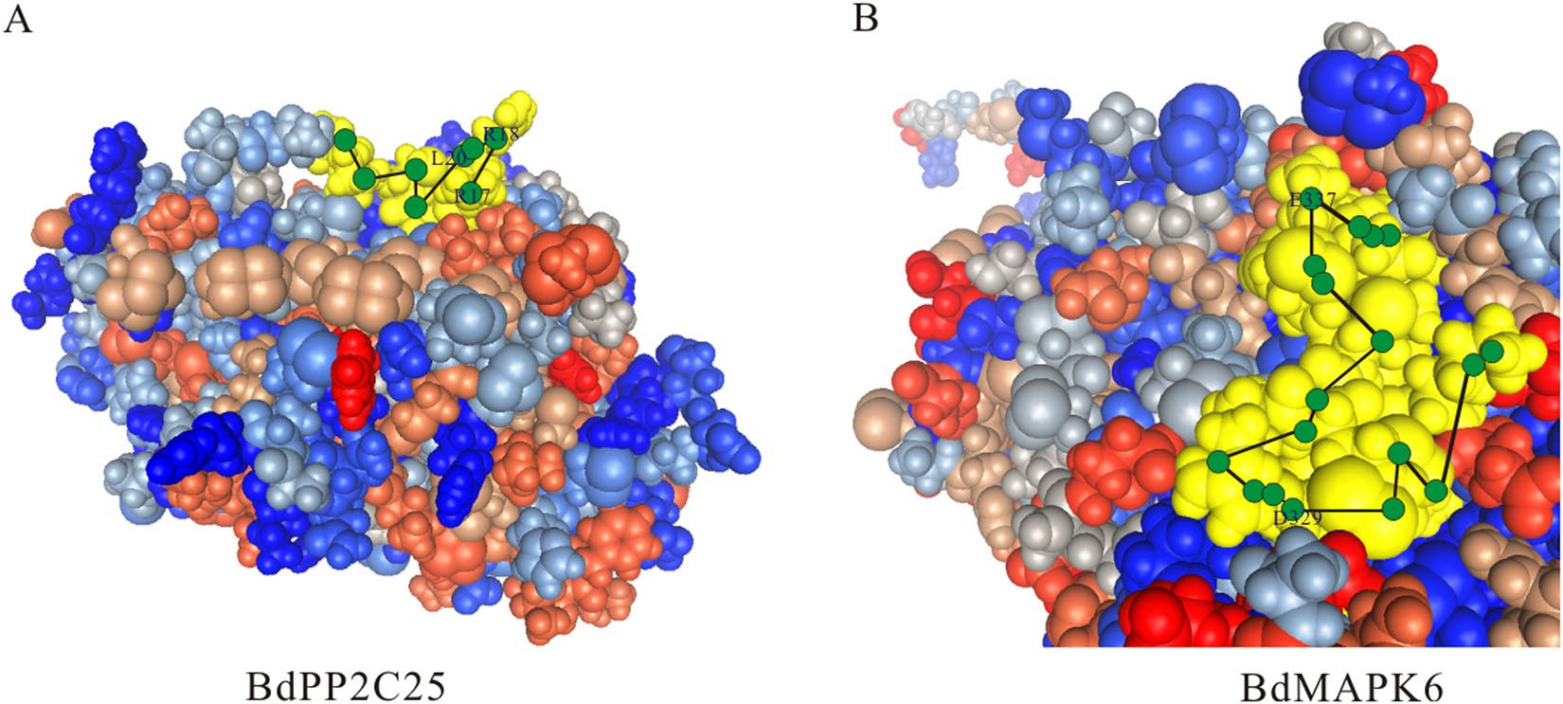
Three-dimensional structural model of the docking site on BdPP2C25 and the CD domain of BdMAPK6. The D-site of BdPP2C25 and CD domain of BdMAPK6 was marked in yellow and connected with the line. Hydrophobic amino acid residues and the basic residues were depicted in blue font; Negative charged amino acid residues are shown in blue type as well.

**Figure 3:**
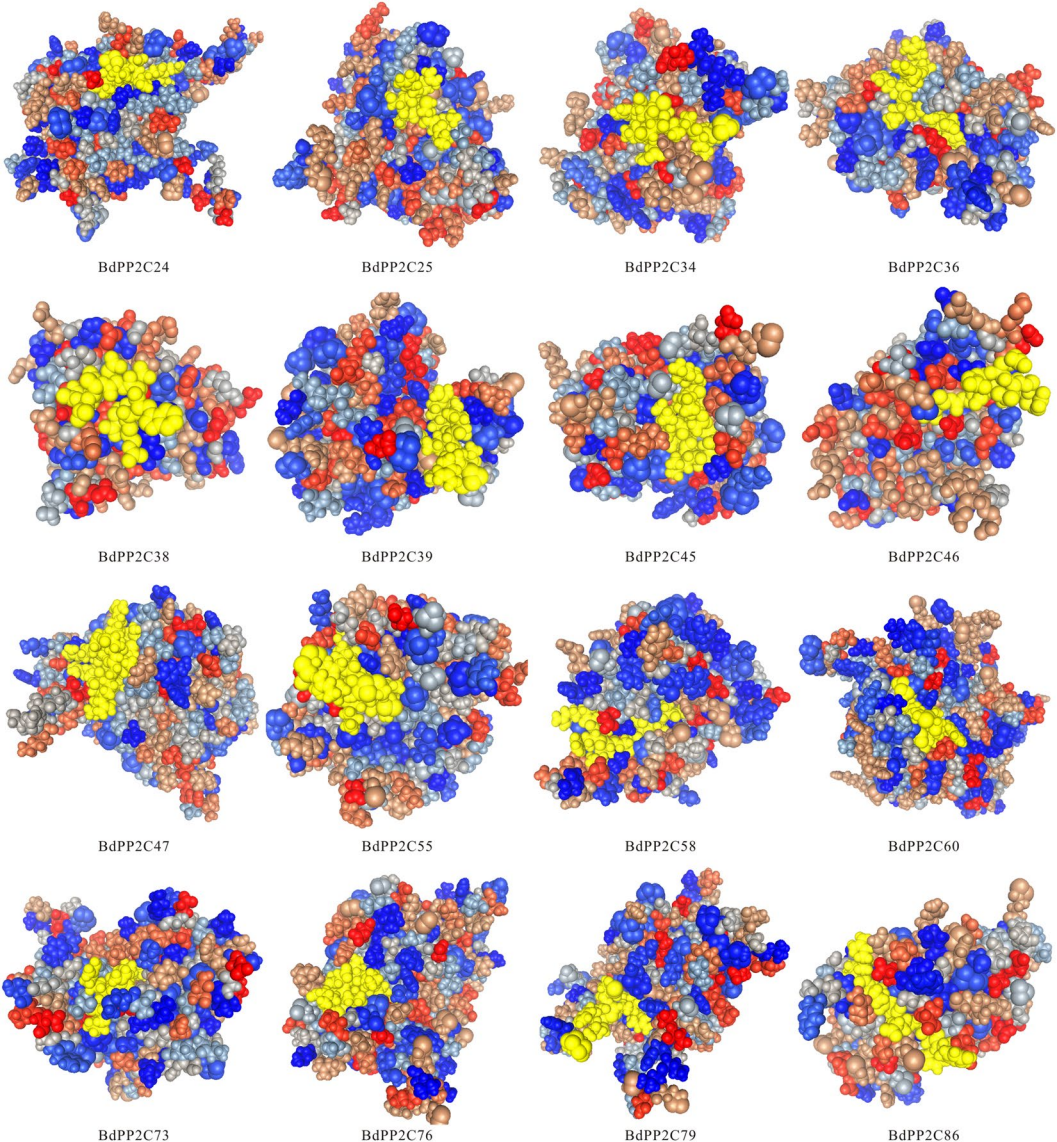
Three-dimensional structural model of the docking site on BdPP2Cs identified interacts with BdMAPKs in this study. The D-site of BdPP2Cs identified interacts with BdMAPKs was marked in yellow.

In order to assess the reliability of prediction results and docking site, we investigated the 3D protein structure of BdMAPK6 in complex with docking peptides from BdPP2C25 (high affinity site, BdPP2C25: **RR**SLSCKA; BdMAPK6, KMLTFDPRQRITVEGAL-visible regions marked in yellow; Fig. 2). It has reported that AtMAPK6 and AtMAPK4 can be dephosphorylated by AtPP2C1 which is orthologous genes with BdPP2C6^38^. Therefore, we choose them to evaluate the possibility of PPIs between BdPP2Cs and BdMAPKs, which was also used as the criterion of cutoff. It is found that the position of BdPP2C6 in “*φ*-X-*φ*” locate in protuberant edge which is easier to insert into the hydrophobic pockets. Moreover, BdMAPK6 in common docking motif has three side chain docking pockets which are consistent with the interaction situation (Fig. 2). The results suggested that the PPI of BdMAPK6 and BdPP2C25 have structural evidence support. Furthermore, other BdPP2C proteins also show similar structure in docking site for BdMAPKs (Fig. 3). For example, the 3D structure of BdPP2C47 harbors a D-site (consensus sequence: RRRRRLEMRRFRL, Table 1), which are consistent with the situation of interacting BdMPKs (Fig. 3).

### Database Validation

To better verify our prediction method, the AVLE of more PPIs with reported in public STRING database were performed. Because these PPIs about PP2C and MAPK have confirmed through experiments, instead of pure untested computational procedure. We can assess the reliability of our method through comparing the difference of AVLE about phosphatase-MAPK interaction between *B. distachyon* and other species. Interestingly, the results show that the AVLE of most (35 pairs) Arabidopsis and yeast PPIs less than 65.74, except 4 pairs PPIs (Table 2). For example, the AVLE of AP2C4-AtMPK6 and AP2C1-AtMPK3 is 73.9 and 70.22, respectively (Table 2). These results suggested that the PPI of BdMAPKs and BdPP2Cs have more favorable evidence support. Because we have already chosen the max AVLE between subgroup B BdPP2Cs and BdMPK3, BdMPK4 or BdMPK6, when the AVLE of most Arabidopsis and yeast PPIs in public database less than 65.74, which indicated that the bigger AVLE of BdPP2C and BdMAPK should even more have protein-protein interaction. That is, these results indirectly suggested that our prediction method have low false positive rate and dependability. Furthermore, in spite of having difference in other species, the same values remain have certain reference value for predicting the PPIs between PP2Cs and MAPKs on other species.

**Table 2:** Validation of our method was used by the PPIs between PP2C and MAPK of Arabidopsis and yeast in STRING database.

MPK_name	MPK_ID	PP2C_name	PP2C_ID	Group	AVLE	Verification
AtMPK3	AT3G45640	AP2C1	AT2G30020	B	70.22	phosphatase activity
AtMPK4	AT4G01370	AP2C1	AT2G30020	B	52.84	BiFC
AtMPK6	AT2G43790	AP2C1	AT2G30020	B	60.56	BiFC,Co-IP, phosphatase activity
AtMPK3	AT3G45640	AP2C2	At1g07160	B	47.95	BiFC
AtMPK4	AT4G01370	AP2C2	At1g07160	B	54.23	BiFC
AtMPK6	AT2G43790	AP2C2	At1g07160	B	47.44	BiFC
AtMPK3	AT3G45640	AP2C3	AT2G40180	B	50.18	Y2H,BiFC,Co-localization
AtMPK4	AT4G01370	AP2C3	AT2G40180	B	56.21	Y2H,BiFC,Co-localization
AtMPK6	AT2G43790	AP2C3	AT2G40180	B	50.53	Y2H,BiFC,Co-localization
AtMPK3	AT3G45640	AP2C4	At1g67820	B	34.48	BiFC
AtMPK6	AT2G43790	AP2C4	At1g67820	B	73.9	BiFC
AtMPK9	AT3G18040	AtPP2C31	AT2G40860	G	67.82	—
AtMPK8	AT1G18150	AtPP2C31	AT2G40860	G	64.08	—
AtMPK6	AT2G43790	AtPP2C31	AT2G40860	G	47.02	—
AtMPK20	AT2G42880	AtPP2C31	AT2G40860	G	50.75	—
AtMPK19	AT3G14720	AtPP2C31	AT2G40860	G	67.7	—
AtMPK18	AT1G53510	AtPP2C31	AT2G40860	G	57.53	—
AtMPK17	AT2G01450	AtPP2C31	AT2G40860	G	46.61	—
AtMPK16	AT5G19010	AtPP2C31	AT2G40860	G	49.32	—
AtMPK15	AT1G73670	AtPP2C31	AT2G40860	G	63.38	—
AtMPK13	AT1G07880	AtPP2C31	AT2G40860	G	32.79	—
AtMPK9	AT3G18040	AtPP2C19	AT2G20050	L	59.5	—
AtMPK8	AT1G18150.	AtPP2C19	AT2G20050	L	58.51	—
AtMPK7	AT2G18170	AtPP2C19	AT2G20050	L	44.64	—
AtMPK6	AT2G43790	AtPP2C19	AT2G20050	L	46.23	—
AtMPK5	AT4G11330	AtPP2C19	AT2G20050	L	42.22	—
AtMPK4	AT4G01370	AtPP2C19	AT2G20050	L	31.87	—
AtMPK3	AT3G45640	AtPP2C19	AT2G20050	L	59.19	—
AtMPK20	AT2G42880	AtPP2C19	AT2G20050	L	45.17	—
AtMPK19	AT3G14720	AtPP2C19	AT2G20050	L	44.14	—
AtMPK18	AT1G53510	AtPP2C19	AT2G20050	L	50.16	—
AtMPK17	AT2G01450	AtPP2C19	AT2G20050	L	60.65	—
AtMPK16	AT5G19010	AtPP2C19	AT2G20050	L	55.98	—
AtMPK15	AT1G73670	AtPP2C19	AT2G20050	L	55.94	—
AtMPK14	AT4G36450	AtPP2C19	AT2G20050	L	34.36	—
AtMPK13	AT1G07880	AtPP2C19	AT2G20050	L	29.38	—
AtMPK10	AT3G59790	AtPP2C19	AT2G20050	L	41.65	—
Hog1	/	PTC1	/	/		Immunoblot
Hog1	/	PTC2	/	/	35.41	Immunoblot, phosphatase activity
Hog1	/	PTC3	/	/	50.61	Fluorescence microscopy

— :other methods.

## Discussions

### D-sites variants for MAPK on PP2Cs in *B. distachyon*

**Figure 4:**
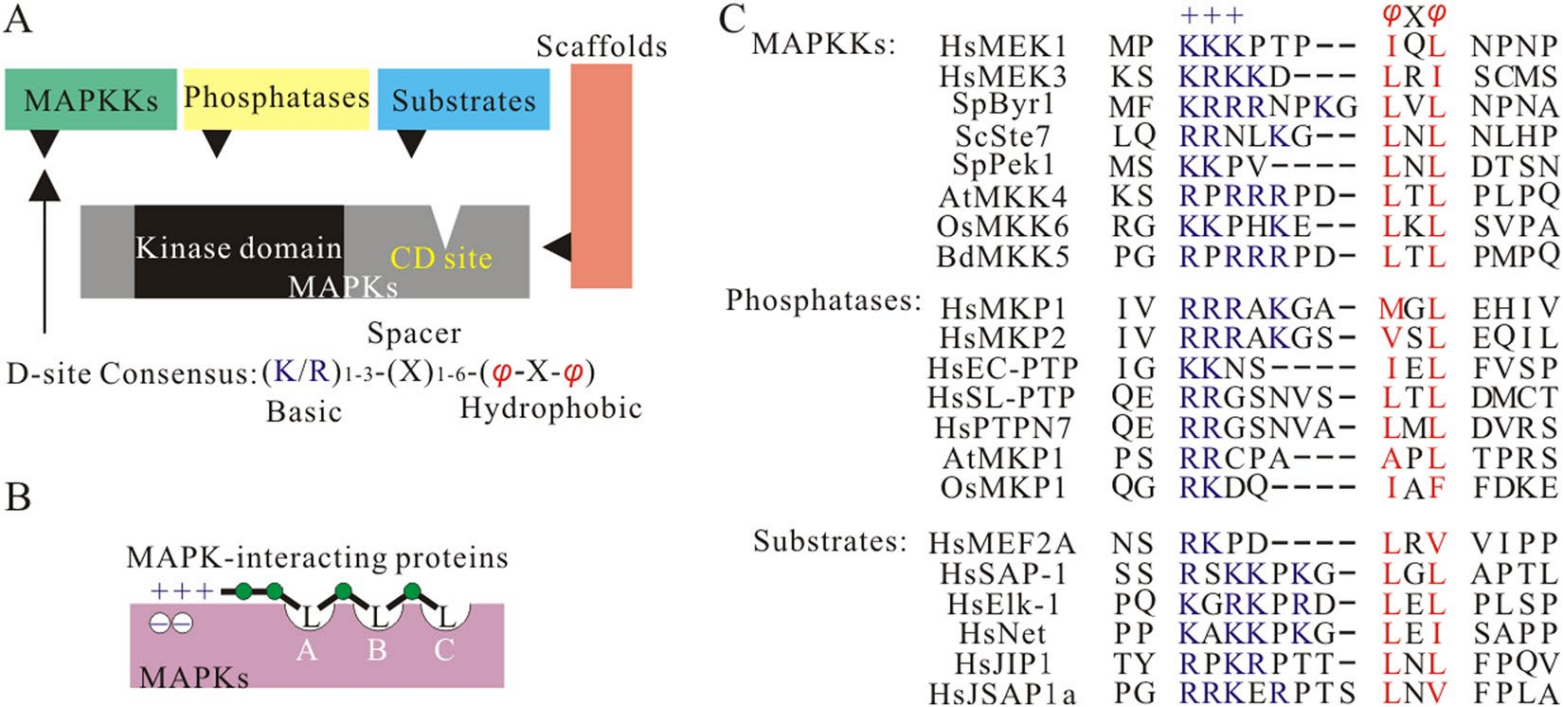
MAP kinases interact with D-sites on substrates and regulators. (**A**) MAPK and several classes of MAPK-interacting proteins. The D-site on MAPK-binding proteins is shown as a triangle. (**B**) Cartoon of MAPK docking surface MAPK and its interaction proteins complex. The peptides display similar interactions with the two main regions of the MAPK: the hydrophobic groove, which has three side chain docking pockets (**A**, **B** and **C**; **B** and **C** were earlier described as “-x- groove”), and the acidic region, known as the “common docking” (CD) site, which binds the basic residues at the N terminus of the docking motifs. (**C**) Comparison of D-sites found in the human, yeast and plant MAPK-interacting proteins. Residues comprising the basic submotif (+++) are shown in blue type; residues comprising the hydrophobic sub-motif (-X-) are shown in red type. Gaps have been introduced to maximize alignment of functionally similar residues; spaces are for visual clarity.

It has been demonstrated that MAPK phosphatases can act as negative regulators of multiple distinct MAPK pathways according to many studies^39^. It is one of key problems to understand their roles on signaling under given conditions how they manage to act specifically on the appropriate substrate. In this context, it is critical to know the mechanisms that mediate the interaction between PP2Cs and MAPKs. In general, MAPK-interaction proteins harbor D-sites at the N-terminus region, which can specific bind cognate CD domain of MAPK(Fig. 4B)^11,12^. The D-site is characterized by a cluster of positively charged amino acids outside the catalytic domain, whose consensus sequence is (R/K_1–3_-X_1–6_-*φ*-X-*φ*) (Fig. 4A,C)^10^. As expected, PP2C-type phosphatases were identified a region matching the consensus MAPK interaction motif (named KIM; [K/R]_(3–4)_-X_(1–6)_-[L/I]-X-[L/I]) in subgroup B phosphatases^38^. BdPP2C6, BdPP2C25, BdPP2C73 and BdPP2C78 belong to the subgroup B PP2Cs, which also has a typical KIM^40^. In addition, it only has reported that the interactions and structural features of PP2Cs with MAPKs in model plant Arabidopsis. So, we also independently investigated the AVLE of PPIs with BdMPK3/4/6 for the clues of their interaction each other (Table 3). However, other PP2Cs maybe interact with MAPK, although the PP2C protein does not apparently contain the KIM on N-terminal extension, commonly used MAPK docking sites. It is possible that these plant PP2C-type phosphatases are also interacted with MAPK through other residues located within or outside the typical D-site (Table 1). Indeed, another type of D-site named DEF motif which has a consensus FXFP of human and yeast PP2C proteins has been reported^41^. Furthermore, spatial and temporal regulators of PP2C dephosphorylation are probably needed to ensure specific downstream activation of MAPKs. Future identification of all PP2C D-sites and their effects to interaction possibility will be necessary for a detailed understanding of PP2C regulation.

**Table 3:** The AVLE of 12 pairs PPIs between BdMAPKs (BdMPK3/4/6) and subgroup B BdPP2Cs.

MPK_name	MPK_ID	PP2C_name	PP2C_ID	PP2C_group	AVLE
BdMPK3	Bradi1g65810	BdPP2C6	Bradi1g16630	B	60.33
BdMPK4	Bradi3g32000	BdPP2C6	Bradi1g16630	B	50.23
BdMPK6	Bradi1g49100	BdPP2C6	Bradi1g16630	B	62.83
BdMPK3	Bradi1g65810	BdPP2C25	Bradi1g65520	B	43.51
BdMPK4	Bradi3g32000	BdPP2C25	Bradi1g65520	B	45.07
BdMPK6	Bradi1g49100	BdPP2C25	Bradi1g65520	B	65.74
BdMPK3	Bradi1g65810	BdPP2C73	Bradi4g21510	B	41.79
BdMPK4	Bradi3g32000	BdPP2C73	Bradi4g21510	B	47.49
BdMPK6	Bradi1g49100	BdPP2C73	Bradi4g21510	B	59.1
BdMPK3	Bradi1g65810	BdPP2C78	Bradi4g40490	B	44.1
BdMPK4	Bradi3g32000	BdPP2C78	Bradi4g40490	B	49.9
BdMPK6	Bradi1g49100	BdPP2C78	Bradi4g40490	B	53.52

### The PPI network of BdPP2Cs and BdMAPKs

To date, knowledge about the function of MAPK signaling pathways is rather limited, especially their negative regulator, including PP2Cs. OsMKP1, a dual-specificity phosphatase, can negatively regulate the OsMPK6 via dephosphorylation to coordinate the trade-off between grain number per panicle and grain size^42^. The expression of AtPTP1, a protein Tyr phosphatase, was manifold increased under salinity stress while decreased obviously in cold stress^43^. DOG1 can interact with the type 2C protein phosphatases AHG1 and AHG3 regulated the ABA signaling pathway to control seed dormancy^36^. Furthermore, little or no information is available on the molecular regulation network about MAPK cascades including activators or regulators. In addition, previous searching for MAPK target network using protein microarrays yielded 570 MPK phosphorylation substrates^44^ and MKKK-MKK-MPK molecular regulation network using co-expression systems showed some signaling pathways^45^. Moreover, despite a large amount of computational approaches have been developed to effectively and accurately predict protein interactions^30,32^, to date we still need develop more effective methods to investigate the PPIs situation of them. However, construction of a PPI network is the first step for the systematically study of a given organism, which not only provides clues for the signaling pathway but also contributes to comprehend protein functions^46^. Therefore, we took a high-throughput approach to understand the PP2C/MAPK signaling pathways. Specially, we identified 96 pairs PPIs and generated a PPI network (Fig. 1). Moreover, our study uncovers a series of putative docking site variants on PP2Cs (Table 1), thus inferring and predicting their functions and roles in MAPK signaling pathways.

### Comparison with other computational methods

So far, a variety of computational methods for predicting PPIs have been developed by investigators. They all can accurately predict protein interaction only based on different aspects. Such as, the evolutionary information, protein structure, physicochemical characteristics, weighted sparse representation, and so on. The incorporating evolutionary information and physicochemical characteristics feature extraction method for predicting PPIs using a newly developed discriminative vector machine (DVM) classifier had proposed^31^. The RVM-BiGP that combines the relevance vector machine (RVM) model and Bi-gram Probabilities (BiGP) for PPIs detection from protein sequences had developed using protein evolutionary information^47^. Moreover, the combining weighted sparse representation based classifier (WSRC) and global encoding (GE) of amino acid sequence had also performed^33^. It has reported a method for inference of protein-protein interactions from protein amino acids sequences^32^. While we also propose a novel computational method for predicting PPIs based on the energy of the protein 3D structure closed. Subsequently, we verified the feasibility of our method through a series of models, including the 3D structure reconstruction, putative protein docking domain prediction and public STRING database validation. For example, BdMAPK6 in common docking motif has three side chain docking pockets which are consistent with the situation of interacting BdPP2C25 which contain a D-site (Fig. 2). The analysis results indicated that our method is feasible for predicting PPIs, and can be used as an effective supplementary tool for future proteomics research in the traditional experimental methods.

## Conclusion

In this study, we proposed in silico “Docking” strategy to predict the PPIs network about between 16 BdMAPKs and 86 BdPP2Cs. Docking studies showed 96 pairs of probable PPIs including all BdMAPK and 49 BdPP2C, which indicated that BdPP2C may be involved in other signaling pathway. In addition, 34 BdPP2C proteins harbor D-site in identified interaction with BdMAPK, which means that D-site plays important role in BdPP2C protein bind to BdMAPK. Moreover, we evaluate the reliability of prediction results and docking site by compare the 3D protein structure of BdMAPK6 and BdPP2C25 which can provide the structural support for our study. Furthermore, STRING database also indicated that our prediction results have low false positive rate and dependability. These findings provide insight into docking interactions in MAPK signaling networks and should be related to efforts to predict new MAPK substrates and regulators by in silico-prediction methods.

## Methods

### Prediction of PPIs between BdPP2Cs and BdMAPKs

**Figure 5:**
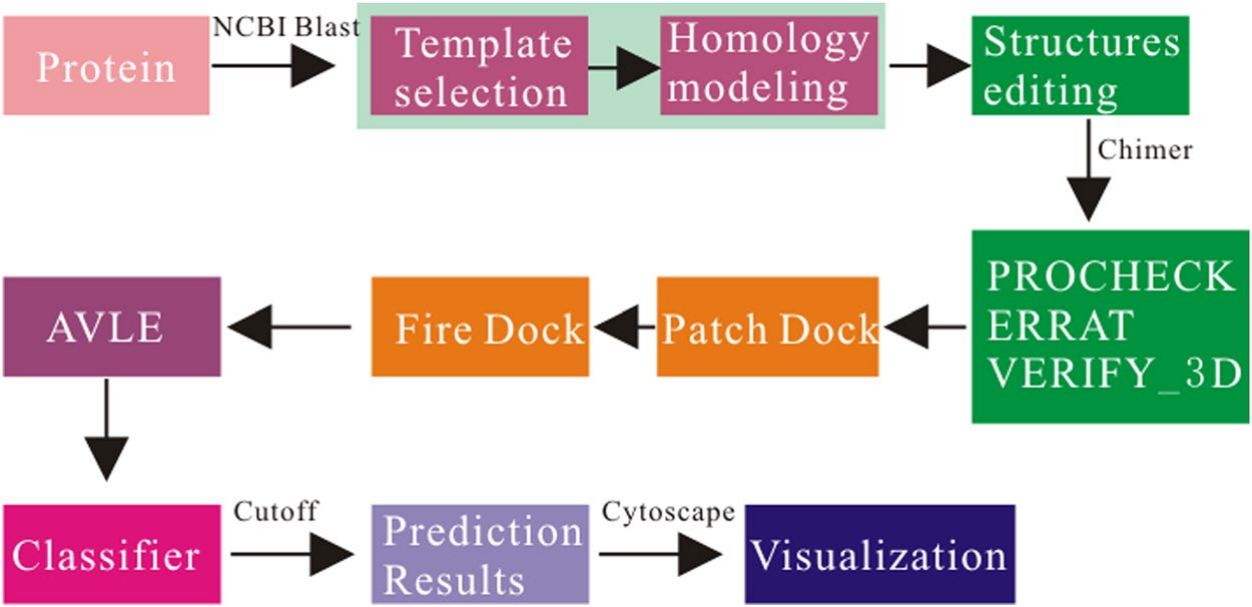
The flow chart of the prediction method of PPIs we proposed.

The sequences of *B. distachyon* (BdMAPKs and BdPP2Cs) were downloaded from PLAZA (https://bioinformatics.psb.ugent.be/plaza/)^48^ in FASTA format. Homology modeling was performed with the help of MODELLER and NCBI BLAST. For reconstructing the 3D conformation of all investigated in this study, a template for homology modeling was searched with PDB search in NCBI BLAST. Six structures were generated in Protein Data Bank proteins for each protein molecule, out of which the minimized average models with maximum score, lowest E-value and with a cut off identity of >30% were selected. The final structures generated by MODELLER 9.17 were used to construct and evaluate 3D models. Structures editing through energy minimization model was implemented using structure minimization tool from enhanced UCSF Chimera (http://www.rbvi.ucsf.edu/chimera/) which was a program for the analysis of molecular structures and related data and interactive visualization using a toolkit Opal^49^. Therefore, the default parameter value of all structures are retained, in details as follows: Steepest descent steps: 100, Steepest descent step size(A): 0.02, Conjugate gradient steps: 10, Conjugate gradient step size: 0.02, Update interval: 10, Fixed atoms: none, Method: also consider H-bonds(slower), Standard residues: AMBER ff14SB, Other residues: AM1-BCC Protonation states for: histidine, Residue-name-based (HIS/HID/HIE/HIP = unspecified/delta/epsilon/both)^50^. The minimized energy structures were finally saved as *.pdf files which were checked and validated by PROCHECK, ERRAT and VERIFY_3D. Subsequently, the refined structures of BdMAPKs were taken as receptor and the structures of BdPP2Cs were taken as ligand for the docking studies on the online Patch Dock server which is based on shape complementary principle and keeping parameter value Clustering RMSD:4.0, Complex Type: Default^51,52^. The results obtained were refined using Fire Dock online server which rearranges the interface side chains and adjusts the relative orientation of the molecules^53^. The AVLE is in solutions number 1000. At last, we can obtain the AVLE of all phosphatase-MAPK interaction pairs (Table S2). The prediction framework is shown in Fig. 5.

### Selection of threshold as a criterion

To distinguish the interacting and non-interacting partners between BdMAPKs and BdPP2Cs, we need chose a reasonable value as a criterion. It has previously reported that phosphatase-MAPK interaction is only found in subgroup B PP2Cs for putative D-site with several kinds of MPKs, including MPK3, MPK4 or MPK6^35,38^. It is more important that these interactions with MPK3, MPK4 and MPK6 depends on D-site in PP2C, which have proved through experiments such as yeast two hybrid screen (Y2H), BiFC assays and so on. According to the principle that homologous genes have similar functions, we therefore analyzed the AVLE of the interaction between all the subgroup B PP2Cs (BdPP2C6, BdPP2C25, BdPP2C73 and BdPP2C78) and BdMAPKs (BdMAPK3, BdMAPK4 and BdMAPK6). Specifically, the AVLE of the interaction between BdPP2C6 and BdMAPKs (BdMAPK3, BdMAPK4 and BdMAPK6) is 60.33, 50.23 and 62.83, respectively (Table 3). Likewise, the AVLE of between BdPP2C25 (43.51, 45.07 and 65.74), BdPP2C73 (41.79, 47.49 and 59.1) and BdPP2C78 (44.1, 49.9 and 53.52) and BdMAPKs are obtained, respectively (Table 3). For maximum assurance of the accuracy of the prediction results, e.g. the false positive rate, etc. we have chosen the max AVLE (that is 65.74) from them was used as a criterion which was distinguished the interacting and non-interacting partners between BdMAPKs and BdPP2Cs. Since there is a smaller AVLE of phosphatase-MPK interaction pairs that are likely to interact, then which has a greater AVLE that is of course more likely to interact.

### Prediction of docking site in BdPP2Cs using hidden Markov model

The docking interactions are contributed to the efficiency of all the enzymatic reactions and the specificity of molecular recognition. In order to search for the docking site for BdMAPKs in BdPP2Cs, we aligned the full-sequence of BdPP2Cs with others that contains conserved D-site using hidden Markov model (HMM). A profile HMM architecture, composed of linked main, insert, and delete states was performed in the programming language Java to implement the computational analysis and prediction. An HMM model of length 19 was designed to be a minimal prescreen that simply checks for a basic residue followed after a spacer of 1–5 residues by a hydrophobic-X-hydrophobic (as defined above)^54^. D-finder is used to select suitable strings and then assigns a standard HMM Viterbi probability score. The code of D-finder is downloadable from http://dfinder.sourceforge.net as a.zip file that contains the Java files along with the original training set file, a sample testing file, and a README file.

### Confirmation of docking motif and interolog based protein-protein interaction prediction

The docking-based and interolog method uses domain interaction information and sequence similarity to infer the potential PPIs, respectively. If two proteins contain an interacting domain pair, it is expected that these two proteins may interact with each other. While if two proteins have interacting homolog in another organism such as Arabidopsis, it is also thought that these two proteins are conserved interaction^55^. Thus, interologs are homologous pairs of protein interactions across different organisms. To assess the PPIs of BdPP2Cs and BdMAPKs, protein 3D structures are used to check their probability of interaction from spatial conformation, especially docking site.

### Validation of results against STRING database with our methods

To validate the feasibility and reliability of our methods for phosphatase-MAPK interaction, the appraisement about AVLE of PPIs that have confirmed with experiments in STRING database^56^ (https://string-db.org/cgi/input.pl) were performed. Subsequently, we compared the AVLE of other species phosphatase-MAPK interaction, including Arabidopsis and yeast.

## Electronic supplementary material

Supplementary Information
