# Supplementary material for: In silico-prediction of protein–protein interactions network about MAPKs and PP2Cs reveals a novel docking site variants in Brachypodium distachyon
Source: Sci Rep. 2018 Oct 10;8:15083. doi: 10.1038/s41598-018-33428-5 (PMC6180098; doi:10.1038/s41598-018-33428-5)
Supplement: Supplementary file 1 — Supplementary Information [file 41598_2018_33428_MOESM1_ESM.pdf]

In silico-prediction of protein–protein interactions network about MAPKs and PP2Cs reveals a novel docking site variants in *Brachypodium distachyon*

Min Jiang [1, 2, #], Chao Niu [1, 2, 3 #], Jianmei Cao [1, 2], Di-an Ni [3] and Zhaoqing Chu [1,2*]

[1]Shanghai Key Laboratory of Plant Functional Genomics and Resources, Shanghai Chenshan Botanical Garden, Shanghai, China
[2]Shanghai Chenshan Plant Science Research Center, Chinese Academy of Sciences, Shanghai, China
[3]School of Ecological Technology and Engineering, Shanghai institute of Technology, Shanghai, China
# These authors contributed equally to this work.
* Correspondence: Zhaoqing Chu (zqchu@sibs.ac.cn)

Table S1. The interaction pairs of BdPP2Cs and BdMPKs in this study.

PPIs	AVLE	PPIs	AVLE	PPIs	AVLE
PP2C2-MPK21-1	76.1	PP2C34-MPK20-3	65.98	PP2C49-MPK21-1	68.43
PP2C3-MPK20-1	67	PP2C34-MPK20-5	66.09	PP2C50-MPK7-1	70.36
PP2C4-MPK16	69.27	PP2C34-MPK21-2	71.71	PP2C50-MPK20-2	67.25
PP2C4-MPK20-2	74.11	PP2C35-MPK11	83	PP2C50-MPK21-1	76.51
PP2C5-MPK17	72.09	PP2C36-MPK20-2	70.32	PP2C50-MPK21-2	78.75
PP2C5-MPK20-2	77.31	PP2C36-MPK21-2	96.73	PP2C54-MPK20-1	68.86
PP2C6-MPK20-1	74.78	PP2C38-MPK17	66.99	PP2C54-MPK20-4	65.95
PP2C7-MPK20-1	78.36	PP2C39-MPK20-1	76.86	PP2C54-MPK21-1	73.04
PP2C8-MPK14	68.05	PP2C39-MPK20-2	67.51	PP2C55-MPK11	75.35
PP2C11-MPK16	67.87	PP2C39-MPK20-3	68.38	PP2C58-MPK20-4	67.7
PP2C13-MPK14	75.88	PP2C39-MPK20-4	65.97	PP2C60-MPK4	102.76
PP2C13-MPK20-2	70.05	PP2C40-MPK7-1	68.33	PP2C62-MPK14	71.25
PP2C14-MPK6	72.29	PP2C41-MPK11	80.13	PP2C62-MPK20-2	66.77
PP2C17-MPK21-1	70.71	PP2C41-MPK20-4	70.76	PP2C63-MPK7-2	70.31
PP2C17-MPK21-2	75.88	PP2C41-MPK21-2	66.36	PP2C71-MPK11	69.21
PP2C19-MPK17	69.18	PP2C42-MPK21-2	69.49	PP2C71-MPK16	71.77
PP2C21-MPK11	79.43	PP2C44-MPK11	68.06	PP2C71-MPK21-2	67.14
PP2C22-MPK20-4	67.86	PP2C44-MPK20-5	72	PP2C73-MPK20-2	68.28
PP2C22-MPK20-5	76.1	PP2C45-MPK17	68.63	PP2C73-MPK20-5	72.65
PP2C22-MPK21-1	90.01	PP2C45-MPK20-1	72.69	PP2C74-MPK20-2	67.24
PP2C22-MPK21-2	89.64	PP2C45-MPK20-2	70.04	PP2C76-MPK3	76.59
PP2C23-MPK14	73.02	PP2C46-MPK20-1	66.02	PP2C79-MPK6	69.05
PP2C23-MPK20-2	69.42	PP2C47-MPK20-2	67.42	PP2C79-MPK20-2	72.79
PP2C24-MPK3	72.84	PP2C47-MPK21-1	77.23	PP2C80-MPK20-4	69.73
PP2C24-MPK14	81.77	PP2C49-MPK3	69.29	PP2C81-MPK7-2	71.23
PP2C25-MPK6	65.74	PP2C49-MPK6	81.56	PP2C81-MPK20-4	67.95
PP2C25-MPK20-2	70	PP2C49-MPK7-1	79.97	PP2C82-MPK20-1	73.08
PP2C28-MPK16	79.78	PP2C49-MPK14	68.84	PP2C82-MPK20-5	75.1
PP2C28-MPK17	72.9	PP2C49-MPK16	71.74	PP2C84-MPK20-1	75.4
PP2C31-MPK17	80.85	PP2C49-MPK20-1	95.43	PP2C84-MPK20-2	68.14
PP2C31-MPK20-2	71.47	PP2C49-MPK20-4	76.37	PP2C84-MPK20-5	71.19
PP2C31-MPK20-5	80.69	PP2C49-MPK20-5	73.33	PP2C86-MPK20-3	71.97

Table S2. List of the AVLE of 1376 pairs PPIs between BdMAPKs and BdPP2Cs.

	MPK3	MPK4	MPK6	MPK7-1	MPK7-2	MPK11	MPK14	MPK16	MPK17	MPK20-1	MPK20-2	MPK20-3	MPK20-4	MPK20-5	MPK21-1	MPK21-2
PP2C1	46.6	42.66	35.75	48.31	39.92	32.95	42.56	39.86	35.46	38.3	51.74	47.01	37.22	42.02	46.15	29.38
PP2C2	62.4	56.59	62.76	49.94	54.47	56.81	61.09	40.59	61.95	57.86	55.26	43.91	54.64	64.47	76.1	39.27
PP2C3	50.1	49.59	59.5	45.85	55.21	42.37	49.91	59.44	45.73	67	56.53	47.86	50.4	58.47	46.54	54.58
PP2C4	40.3	57.54	52.21	43.08	49.26	45.03	46.35	69.27	55.37	58.28	74.11	36.46	43.28	53.76	39.91	52.52
PP2C5	61.1	46.05	54.61	56.76	49.51	47.64	45.41	46.47	72.09	54.17	77.31	45.01	46.28	44.61	55.1	43.55
PP2C6	60.3	50.23	62.83	50.04	43.77	47.86	48.9	46.14	40.53	74.78	53.93	43.3	51.32	46.14	55.05	59.32
PP2C7	59.7	46.5	55.5	52.48	51.3	55.9	51.53	49.57	53.69	78.36	47.48	40.68	58.83	54.36	54.6	41.78
PP2C8	60.7	46.21	51.47	44.91	54.68	46.32	68.05	40.68	50.61	47.87	53.39	38.17	48.48	63.7	47.91	50.69
PP2C9	50.4	52.83	56.3	56.33	56.38	43.14	48.61	49.07	51.08	52.72	45.7	48.5	46.02	52.83	38.46	49.26
PP2C10	40.9	55.6	45.42	53.71	33.68	47.35	51.64	46.16	42.84	53.46	46.96	43.09	62.73	54.34	45.02	45.46
PP2C11	42.3	42.31	53.47	51.43	50.46	42.27	47.53	67.87	57.55	61.34	62.44	61.4	49.91	53.56	53.61	42.57
PP2C12	50.6	47.08	37.82	46.03	36.41	39.18	45.99	48.88	64.65	47.38	50.24	54.46	37.63	51.12	31.69	31.92
PP2C13	48.8	42.89	39.43	46.67	49.84	45.23	75.88	50.27	57.46	64.83	70.05	57.83	48.35	59.24	55.87	42.55
PP2C14	58	50.4	72.29	50.94	55.46	45.72	46.83	46.64	50.7	45.05	49.42	45.48	34.18	55.02	33.36	40.03
PP2C15	47.3	36.88	51.35	42.5	48.21	42.76	45.06	37.3	53.66	65.37	50.83	52.27	39.64	52.44	34.23	41.26
PP2C16	35.9	41.99	52.12	44.74	65.25	65.31	29.86	43.8	34.38	45.17	43.46	47.85	50.91	54.35	48.98	44.87
PP2C17	49.2	39.04	50.05	52.19	46.16	51.12	48.81	52.53	36.35	52.24	55.89	42.53	40.25	58.86	70.71	75.88
PP2C18	45.2	45.6	52.96	54.35	46.36	49.24	38.83	40.87	41.21	58.82	54.84	46.04	36.95	57.53	54.28	44.13
PP2C19	43.8	31.99	47.96	32.86	49.22	40.77	54.67	51.29	69.18	43.48	46.36	27.09	52.67	48.15	56.86	62.09
PP2C20	36.7	39.59	52.55	41.37	62.67	44.95	46.81	38.92	53.33	45.97	45.15	44.39	60.68	44.09	47.87	49.65
PP2C21	47.3	49.66	59.59	48.52	51.45	79.43	59.49	61.73	44.59	57.23	57.18	52.61	48.34	51.84	43.99	46.98
PP2C22	53.2	41.43	55.5	52.44	50.89	41.68	43.4	43.75	53.89	60.26	61.11	55.12	67.86	76.1	90.01	89.64
PP2C23	57.7	59.43	46.14	45.02	49.91	45.71	73.02	43.42	57.74	50.14	69.42	50.95	57.69	62.96	49.73	43.8
PP2C24	72.8	52.64	61.54	62.14	53.07	61.33	81.77	62.22	44.83	58.19	55.24	51.04	48.63	43.87	52.16	46.91
PP2C25	43.5	45.07	65.74	51.45	45.55	45.61	41.87	50.11	44.98	56.22	70	36.06	49.06	65.19	46.01	43.38
PP2C26	41.2	61.54	41.57	34.66	52.61	44.21	44.28	43.86	63.67	57.46	47.12	31.11	37.67	36.85	53.07	49.53
PP2C27	40.6	45.88	39.64	52.82	40.26	43.17	38.04	59.17	52.24	50.64	42.9	56.04	55.1	44.79	47.92	38.91
PP2C28	52.9	52.99	48.26	50.42	61.56	57.4	53.23	79.78	72.9	51.29	56.85	43.77	47.41	42.99	53.63	46.85
PP2C29	41.7	37.41	44.67	54.24	43.6	53.29	34.31	47.45	57.1	62.27	56.51	44.61	60.14	65.02	57.6	34.42
PP2C30	42.4	50.92	41.34	46.73	61.29	47.25	36.44	40.13	44.86	47.74	43.19	36	47.17	39.25	45.7	34.63
PP2C31	54.5	37.58	54.29	50.73	41.74	54.38	46.21	64.74	80.85	54.67	71.47	45.26	60.45	80.69	61.08	65.3
PP2C32	40.2	47.61	50.36	45.27	46.51	43.85	53.99	45.07	45.6	47.14	62.35	42.49	60.02	61.15	60.04	51.54
PP2C33	60.3	56.13	48.14	43.61	43.69	59.03	49.52	53.34	63.15	46.41	54.91	53.65	46.61	46.29	55.2	64.32
PP2C34	45.7	40.03	44.89	54.47	47.89	42.94	45.11	54.59	57.6	56.94	63.59	65.98	53.33	66.09	62	71.71
PP2C35	45.3	54.83	46.2	50.77	45.1	83	46.46	41	37.12	49.63	46.9	38.85	51.19	40.47	57.17	39.16
PP2C36	60	43.06	50.05	39.74	54.05	40.89	45.06	51.81	44.08	52.87	70.32	40.18	42.39	50.88	49.09	96.73
PP2C37	48	44.81	54.09	41.89	45.55	45.1	44.53	48.19	48.86	45.81	47.54	34.94	40.64	43.5	49.92	46.85
PP2C38	55.9	43.56	55.42	54.66	49.48	55.66	38.43	48.47	66.99	51.08	60.65	54.01	43.46	55.62	44.28	47.24
PP2C39	52.8	47.64	56.46	55.1	50.97	61.17	49.7	58.82	64.61	76.86	67.51	68.38	65.97	57	44.16	58.43
PP2C40	47.9	49.01	46.89	68.33	57.27	51.87	51.23	45.17	47.13	48.42	45.7	54.03	42.52	44.63	44.73	47.22

PP2C41	57.8	51.4	57.57	57.08	59.08	80.13	60.71	56.12	62.37	64	53.25	60.94	70.76	54.09	60.27	66.36
PP2C42	54.6	44.79	56.49	60.98	45.15	53.68	56.5	59.9	61.2	57.05	45.7	47.85	56.89	41.51	51.91	69.49
PP2C43	30.3	37.15	38.62	42.5	60.92	36.06	40.46	51.06	40.9	48.36	52.89	33.12	40.53	37.72	34.27	33.33
PP2C44	63.4	51.09	62.78	50.51	52.95	68.06	43.5	57.74	56.62	51.65	56.97	63.37	52.51	72	55.26	52.71
PP2C45	63.8	51.44	62.51	53.27	52.96	54.52	57.35	49.11	68.63	72.69	70.04	55.99	49.27	51.45	59.97	52.16
PP2C46	45.6	42.83	51.47	45.99	47.82	52.26	40.04	46.86	43.45	66.02	60.05	48.47	43.04	50.29	41.06	42.61
PP2C47	51.7	39.44	47.6	37.78	46.19	56.39	47.2	58.62	55.36	63.32	67.42	35.59	38.71	49.84	77.23	43.64
PP2C48	33.4	46.43	37.27	46.16	47.8	45.58	49.07	48.52	59.26	53.19	62.99	33.8	46.58	46.55	57.51	63.2
PP2C49	69.3	62.45	81.56	79.97	60.67	62.53	68.84	71.74	58.55	95.43	60.31	59.29	76.37	73.33	68.43	64.85
PP2C50	58.1	41.47	54.61	70.36	54.6	56.12	53.72	53.17	56.52	65.6	67.25	58.3	48.46	58.03	76.51	78.75
PP2C51	45.4	35.4	47.03	49.97	41.86	47.47	45.46	41.89	39.79	51.7	38.8	39.28	50.18	41.7	52.85	55.68
PP2C52	43.1	43.22	40.89	51.64	47.75	59.86	50.05	45.69	53.84	58.12	54.99	47	38.56	52.49	44.92	43.11
PP2C53	50.4	52.46	60.31	60.31	62.21	59.15	55.68	56.05	48.14	60.74	58.27	50.32	59.24	49.07	58.3	51.75
PP2C54	54.1	49.79	49.03	57.29	49.96	52.8	54.39	63.54	42.46	68.86	60.05	51.64	65.95	54.74	73.04	58.58
PP2C55	54.1	49.61	48.28	59.27	42.75	75.35	58.28	51.71	37.65	65.31	63.11	45.41	53.27	38.33	47.61	54.29
PP2C56	63.8	46.67	58.82	46.02	53.91	46.86	56.69	57	47.5	56.43	57.25	47.84	57.53	61.45	49.94	58.76
PP2C57	49.7	47.74	50.93	54.67	47.59	42.03	49.2	49.89	44.93	55.69	51.18	60.65	43.49	48.77	55.33	46.98
PP2C58	58.7	47.69	45.87	62.11	61.11	48.45	48.23	61.93	54.47	61.78	61.46	46.79	67.7	55.47	51.75	50.65
PP2C59	42.2	46.5	47.77	42.31	43.44	48.14	50.36	44.68	34.03	47.53	53.37	39.96	46.95	35.6	35.04	46.9
PP2C60	59.3	102.8	53.58	51.59	56.08	60.38	52.87	59.41	51.66	60.43	44.05	63.65	55.65	39.61	51.18	52.58
PP2C61	47.6	53.61	52.68	41.63	43.48	53.77	41.98	44.15	45.68	47.1	58.18	56.12	36.89	56.92	40.47	33.62
PP2C62	47.5	52.05	55.29	51.94	44.44	59.02	71.25	55.46	63.31	56.85	66.77	42.78	49.06	52.33	61.33	55.69
PP2C63	55	57.34	48.3	53.5	70.31	40.64	50.48	54.17	64.98	47.07	59.53	59.98	49.63	50	51.73	42.28
PP2C64	54.7	61.68	51.05	44.86	50.48	65.61	55.62	55.55	46.01	57.75	58.11	45.84	52.27	57.18	53.72	39.74
PP2C65	50.6	41.29	42.36	50.41	51.72	50.86	57.8	42.95	65.63	46.54	50.06	58.45	54.77	38.05	45.57	52.96
PP2C66	49.2	42.66	64.75	43.67	54.57	44.97	61.57	63.59	56.29	51.71	50.17	48.25	63.46	47.55	59.21	51.43
PP2C67	46.2	57.71	50.14	45.6	46.96	53.06	50.59	42.92	38.77	53.27	49.21	38.53	47.01	56.9	53.39	50.14
PP2C68	49.4	56.02	60.41	43.45	64.76	53.22	50.94	45.21	44.46	62.1	58.18	40.45	49.36	51.93	54.08	41.68
PP2C69	53.8	46.2	44.54	38.53	50.06	44.54	35.21	43.5	31.02	42.11	59.58	44.41	55.19	39.91	37.01	56.98
PP2C70	52.7	48.09	50.06	49.49	45.97	56.7	57.06	48.4	50.27	49.67	51.25	43.04	45.41	52.43	44.52	50.22
PP2C71	40.3	53.31	43.21	59.45	58.6	69.21	59.38	71.77	51.19	59.34	56.29	33.98	64.21	62.43	50.65	67.14
PP2C72	53.7	37.61	50.95	43.93	49.08	62.53	49.5	37.37	60.56	47.13	49.45	39.17	53.86	56.72	36.52	38.11
PP2C73	41.8	47.49	59.1	61.96	52.77	54.81	42.36	53.9	46.81	49	68.28	54.48	51.61	72.65	43.19	40.93
PP2C74	55.5	39.1	48.42	43.36	48.59	46.83	54.9	45.66	45.31	54.93	67.24	48.23	55.92	53.52	38.84	58.29
PP2C75	38	48.95	45.29	52.21	44.11	39.1	49.25	39.84	46.64	41.58	49.26	51.27	45.72	36.69	36.62	46.77
PP2C76	76.6	40.93	50.45	52.89	60.36	59.87	53.09	44.46	58.5	50.76	48.23	52.47	53.16	49.77	47.96	46.8
PP2C77	39	38.96	34.75	41.62	37.97	47.13	42.46	48.95	56.04	39.64	52.36	53.57	40.8	38.56	33.72	43.59
PP2C78	44.1	49.9	53.52	56.79	60.2	47.48	39.74	42.76	56.62	59.17	57.87	45.23	51.68	46.19	39.81	55.17
PP2C79	53	55.76	69.05	60.84	53.85	45.08	58.32	43.78	55.25	45.78	72.79	43.44	62.26	51.92	47.27	47.54
PP2C80	60.2	39.97	58.16	58.63	53.68	63.04	52.85	51.41	51.91	61.54	52.5	39.21	69.73	53.67	57.84	48.41
PP2C81	48.4	54.04	52.47	51.7	71.23	52.54	44.87	57.14	47.63	60	65.02	63.92	67.95	50.13	43.9	63.16
PP2C82	54	54.66	50.54	56.61	48.61	49.36	47.81	56.8	65.7	73.08	62.47	55.6	51.47	75.1	60.73	51.94
PP2C83	41.1	46.83	33.26	44.4	48.87	35.65	57.62	46.48	38.38	53.23	46.16	56.46	43.93	43.54	43.06	40.72
PP2C84	51.4	61.19	52.54	54.45	51.16	47.53	59.96	49.63	53.79	75.4	68.14	55.2	65.68	71.19	61.15	61.74

PP2C85	51	51.7	58.29	43.94	45.43	42.06	56.36	50.99	47.07	46.95	57.19	43.93	60.91	47.15	39.36	47.74
PP2C86	59.1	50.32	51.26	53.94	56.31	58.54	59.75	50.38	53.19	52.46	53.53	71.97	44.95	56.9	46.8	51.59
